# Diagnosing sarcopenia and myosteatosis based on chest computed tomography images in healthy Chinese adults

**DOI:** 10.1186/s13244-021-01106-2

**Published:** 2021-11-06

**Authors:** Lingling Tan, Guiyi Ji, Ting Bao, Hongbo Fu, Ling Yang, Ming Yang

**Affiliations:** 1 Center of Gerontology and Geriatrics, West China Hospital, Sichuan University, Chengdu, Sichuan China; 2 Health Management Center, West China Hospital, Sichuan University, Chengdu, Sichuan China; 3 Outpatient Department, West China Hospital, Sichuan University, No 37 Guoxue Lane, Chengdu, 610041 Sichuan China; 4 National Clinical Research Center for Geriatrics, West China Hospital, Sichuan University, No 37 Guoxue Lane, Chengdu, 610041 Sichuan China; 5 Precision Medicine Research Center, West China Hospital, Sichuan University, Chengdu, Sichuan China

**Keywords:** Muscle wasting, Muscle depletion, Skeletal muscle mass, Computed Tomography, Fat infiltration

## Abstract

**Background:**

Measuring muscle mass and muscle quality based on chest Computed Tomography (CT) images would facilitate sarcopenia and myosteatosis research. We aimed (1) to measure muscle mass and myosteatosis based on chest CT images at the 12^th^ thoracic vertebra level and compare the relevant indicators with whole-body skeletal muscle mass (BSM) and whole-body fat mass (BFM) measured by bioelectrical impedance analysis; and (2) to determine the cut-off points of these indicators for diagnosing sarcopenia or myosteatosis in healthy Chinese adults.

**Methods:**

Chest CT images were analyzed using a segmentation software. Skeletal muscle area (SMA), skeletal muscle radiodensity (SMD), and intermuscular adiposity tissue (IMAT) were measured. Skeletal muscle indices (SMIs) and IMAT/SMA ratio were calculated.

**Results:**

We included 569 participants. SMA, SMA/height^2^, and SMA/BMI were strongly and positively correlated with BSM (*r* = 0.90, 0.72, and 0.69, respectively, all *p* < 0.001); whereas SMA/weight was moderately and positively correlated with BSM (*r* = 0.38, *p* < 0.001). IMAT and IMAT/SMA were strongly and positively correlated with BFM (*r* = 0.67 and 0.58, respectively, both *p* < 0.001). SMD was moderately and negatively correlated with BFM (*r* = − 0.40, *p* < 0.001). We suggest SMA/height^2^ (< 25.75 cm^2^/m^2^ in men and < 20.16 cm^2^/m^2^ in women) for diagnosing sarcopenia and SMD (< 37.42 HU in men and < 33.17 HU in women) or IMAT (> 8.72 cm^2^ in men and > 4.58 cm^2^ in women) for diagnosing myosteatosis.

**Conclusions:**

Muscle mass indicators (SMA and SMIs) and muscle quality indicators (SMD, IMAT, and IMAT/SMA) measured by chest CT images are valuable for diagnosing sarcopenia and myosteatosis, respectively.

## Key points

- Chest CT images can be used to assess muscle quantity and quality.
- Sarcopenia can be determined using T12 SMA and T12 SMA/height^2^.
- Myosteatosis can be determined using T12 SMD and T12 IMAT.

## Background

Sarcopenia originally refers to the age-related loss of muscle mass [1]. It has been related to many adverse health outcomes, such as risk of falls, cardiovascular diseases, depression, functional disability, poor quality of life, and even death, in different populations [2–4]. Recently, the updated version of the European Working Group on Sarcopenia in Older People (EWGSOP2), the most widely used sarcopenia guideline, emphasized that muscle quality played an important role in the defining feature of sarcopenia [3].

Muscle quality refers to “micro- and macroscopic changes in muscle architecture and composition” [3]. Fat infiltration in skeletal muscle, also known as myosteatosis, is one of the most widely used indicators for assessing muscle quality [5]. Myosteatosis includes three components: (1) intramyocellular lipids (IMCL); (2) intramuscular adipose tissue (IntraMAT), the extracellular fat within an individual muscle; and (3) intermuscular adipose tissue (IMAT), the extracellular fat beneath the fascia and between muscle groups [5]. Myosteatosis was also found to be associated with adverse outcomes, such as muscle weakness, functional disability, and death [5–10].

Both Magnetic resonance imaging (MRI) and Computed tomography (CT) are suggested to measure muscle mass and myosteatosis [3]. CT is a promising imaging tool for estimating muscle mass via measuring skeletal muscle cross-sectional area (SMA). Additionally, CT can identify myosteatosis not only by directly measuring IMAT but also by indirectly estimating IntraMAT and IMCL via measuring skeletal muscle radiodensity (SMD) expressed in Hounsfield Unit (HU) [5]. The lower the HU, the lower the radiodensity, and the higher the degree of IntraMAT and IMCL [5]. Evaluating whole-body muscle mass and myosteatosis through a whole-body CT scan is supposed to be accurate and reliable, but this is not practical due to high cost and radiation exposure. A more practical method is to measure muscle mass and myosteatosis based on clinically acquired CT scans for the management of other diseases [11]. Therefore, choosing an optimal level of CT axial images to represent whole-body muscle mass or myosteatosis becomes crucial [12]. The 3^rd^ lumbar vertebra (L3) is the commonly used CT level for measuring muscle mass and myosteatosis. However, abdominal CT is not commonly used in clinical practice. In contrast, chest CT is far more widely used in clinical practice. Therefore, if muscle mass and myosteatosis can be evaluated via chest CT images, the sarcopenia and myosteatosis research based on CT images would be significantly facilitated.

A recent study found a strong correlation between muscle mass measured by chest CT at the 12^th^ thoracic vertebra (T12) and that measured by abdominal CT at the L3 level (*r* = 0.724, *p* < 0.001) [13]. Another recent study in *JAMA surgery* found that CT-defined sarcopenia at the T12 level was associated with one-year mortality in older trauma patients [14]. However, there is currently no study assessing myosteatosis based on chest CT images. Thus, we conducted this study (1) to measure muscle mass and myosteatosis based on chest CT images at the T12 level and to compare the relevant indicators with whole-body muscle mass and fat mass measured by bioelectrical impedance analysis (BIA); and (2) to determine the cut-off points of these indicators for diagnosing sarcopenia and myosteatosis in a study population of healthy Chinese adults.

## Materials and methods

### Study population

From August 2019 to July 2020, we continuously invited healthy adults (aged 18 years and older) who received routine health examinations in West China Hospital, Sichuan University, to participate in this study. The exclusion criteria were: (1) individuals who did not receive a chest CT scan; (2) individuals with chronic diseases, such as hypertension, diabetes, coronary heart disease, stroke, chronic respiratory diseases, liver diseases, gastrointestinal diseases, and any type of tumor; (3) individuals with low-quality CT images or had any anatomical distortion such as chest wall edema or loss of any muscle mass area on CT images; (4) individuals with planted pacemaker; and (5) individuals with visible edema. The clinical information collection, anthropometry measurements, and blood samplings were performed on the same day by trained nurses. The study protocol was approved by the Biomedical Ethics Committee of West China Hospital, Sichuan University. All participants signed the written informed consent.

### Measurement of anthropometry and laboratory parameters

The following information was collected from the Electronic Health Record System: age, sex, smoking status, and alcohol drinking status. Waist circumference (WC) was measured at the mid-point between the costal inferior border and the iliac crest in a horizontal plane using a flexible rule to the nearest of 0.1 cm. Body height and weight were measured using an automatic body scale (Sonka Co., Ltd, Shenzhen, China) to the nearest of 0.1 cm and 0.1 kg, respectively. Body mass index (BMI) was calculated using the equation: BMI = weight (kg) / height^2^ (m^2^).

After at least 8 h fasting, early morning blood was drawn from an antecubital vein in the arm of each participant. Levels of fasting total cholesterol, triglycerides, high-density lipoprotein-cholesterol (HDL-C), and low-density lipoprotein-cholesterol (LDL-C) were tested using the enzymatic colorimetric method with the Toshiba 200FR Neo analyzer (Toshiba Medical System Co., Ltd., Tokyo, Japan).

### Measurement of body composition based on CT images

Chest CT scans were completed on the same day of anthropometry measurements for each participant using a 16-slice spiral CT scanner (Brilliance; Philips Healthcare, Ohio, USA) with a 5-mm slice thickness. Acquisition parameters were as follows: 100–140 kV, variable mAs based on the patient’s body size, and detector collimation of 0.75–1.5 mm.

Unenhanced cross-sectional CT images at the T12 level were analyzed using a dedicated segmentation software (Mimics version 21.0; Materialise, Leuven, Belgium). On a single CT image, skeletal muscle area (SMA) was segmented according to the widely accepted muscle tissues thresholds (− 29 to 150 HU) [15], including *erector spinae, latissimus dorsi, rectus abdominis, obliquus externus, internus abdominis*, and *internal and external intercostal muscles*. The mean of skeletal muscle radiodensity (SMD) of T12 SMA was automatically calculated by Mimics software. The lower the SMD, the higher the degree of myosteatosis. Furthermore, IMAT was segmented according to the widely accepted fat tissue thresholds (− 30 to − 190 HU) [10]. Myosteatosis severity increases with increased IMAT. A trained researcher (L.T.) who was blinded to the participants’ clinical information segmented all CT images, and another researcher (G.J.) reviewed the segmented images.

According to previous studies [16, 17], we divided SMA by body height squared (m^2^), body weight (kg), and BMI (kg/m^2^), respectively, to adjust for the impact of body size on SMA. The height-, weight-, and BMI-adjusted SMAs were collectively known as skeletal muscle indices (SMIs). As reported previously [18], IMAT/SMA ratio was also calculated using the equation: IMAT/SMA ratio = IMAT (cm^2^)/ SMA (cm^2^) × 100%.

### Body composition measurements based on BIA

Whole-body skeletal muscle mass (BSM) and whole-body fat mass (BFM) were estimated by segmental multi-frequency BIA (InBody 770, Biospace Co., Ltd., Korea). After at least 8 h fasting, participants were asked to stand on the platform of the BIA device barefoot with their feet on the electrode and to grasp the handles of the device with their fingers directly contacting with the electrodes. Then, they were asked to stand still for 1 min with their elbows fully extended, and their shoulder abducted to approximately 30°. The device measured the bioimpedance of the participant’s body and estimated BSM and BFM automatically.

### Statistical analysis

Continuous data are presented as mean and standard deviation (SD) or median and interquartile range where appropriate; whereas categorical data are presented as number and percentage. The differences between groups were compared using independent samples t-test or Mann–Whitney U test for the continuous variables with normal or abnormal distribution, respectively. The distributions of SMA, SMD, IMAT, and IMAT/SMA in men and women were presented in density plots.

Due to the significant differences in SMA, SMIs (SMA/height^2^, SMA/weight, and SMA/BMI), SMD, and IMAT between men and women, we stratified the data by sex. Pearson’s correlation coefficients (r) were calculated to explore the correlations of SMA and SMIs with BSM. Spearman’s rank correlation coefficients (ρ) were calculated to explore the correlations of SMD, IMAT, and IMAT/SMA with BFM. We also used scatter plots and linear models to examine the correlation between SMA and BSM and the correlations of SMD and IMAT with BFM. The correlation coefficients are considered as high, moderate, or low when r (or ρ) is > 0.5, 0.3–0.5, or < 0.3, respectively [19].

We defined the T-score for SMA and SMIs by calculating the difference between the individual’s measured SMA (or SMIs) and the corresponding means of healthy young adults (aged 18 to 40 years). The equation for T-score calculation is as follows: T-score = (individual’s value—young adults’ mean value)/young adults’ SD value. According to the EWGSOP2 [3], individuals with an SMA (or SMIs) less than the sex-specific mean values of SMA (or SMIs) at the point of T-score = − 2.0 in the young reference group were considered to have sarcopenia. As previously reported [17, 20], we also provided the sex-specific mean values of SMA (or SMIs) at the point of T-score = − 1.0 or − 2.5 in the young reference group as the alternative cut-off points of sarcopenia.

Because SMD, IMAT, and IMAT/SMA were of abnormal distribution, SMD-defined myosteatosis was determined when the individual’s SMD was less than the sex-specific fifth percentile (p5) cut-off points of SMD in the young reference group. The sex-specific first percentile (p1) and 10^th^ percentile (p10) cut-off points of SMD were also reported. Similarly, IMAT-defined myosteatosis was determined when the individual’s IMAT was more than the sex-specific 95^th^ percentile (p95) cut-off points of IMAT in the young reference group. The sex-specific 90^th^ percentile (p90) and 99^th^ percentile (p99) cut-off points of IMAT were also reported.

All statistical analyses were performed in SPSS software 26.0 (IBM SPSS Inc., New York, US) and R version 3.5.1(R Foundation for Statistical Computing, Vienna, Austria). A *p* value < 0.05 indicates statistical significance.

## Results

### Study population

**Fig. 1:**
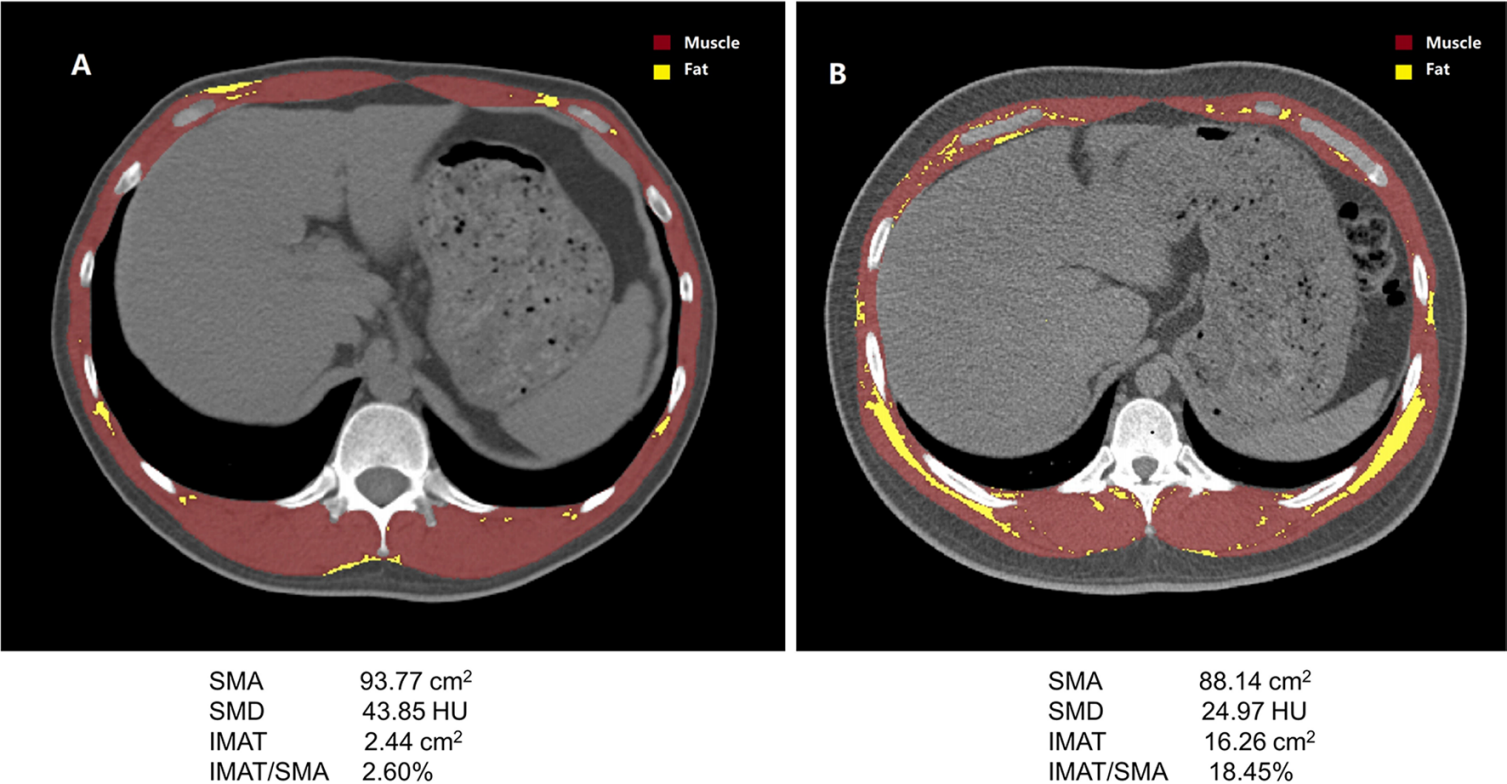
Cross-sectional CT images at the T12 level used for the quantification of muscle mass and fat mass in a 39-year-old man (**A**) and a 62-year-old woman (**B**)

**Fig. 2:**
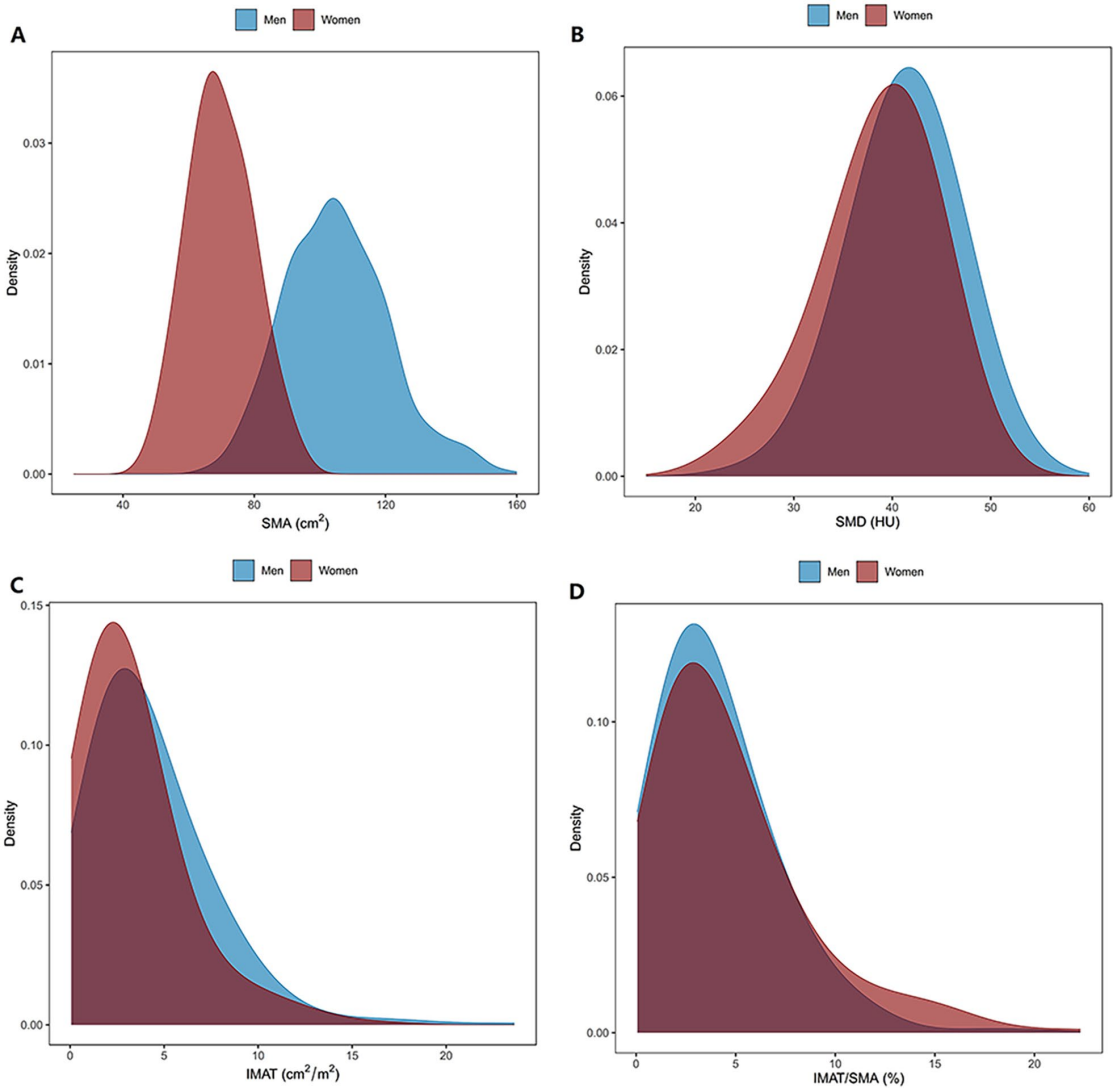
Density distribution of SMA (**A**), SMD (**B**), IMAT (**C**), and IMAT/SMA (**D**)

A total of 569 subjects (359 men and 210 women) were included in this study. Table 1 shows the baseline characteristics of the total study population and the young reference group. Not surprisingly, the BSM, BFM, SMA, SMIs, SMD, and IMAT were significantly higher in men than in women. However, there is no significant difference among men and women concerning IMAT/SMA. The density distributions of SMA, SMD, IMAT, and IMAT/SMA of men and women are shown in Figs. 1, 2.

**Table 1:** Baseline characteristics of the study population and the young adults

	Whole Study Population			Young reference group		
	Men (n = 359)	Women (n = 210)	p	Men (n = 142)	Women (n = 102)	p
Age (years)	43.15 ± 10.67	41.47 ± 11.03	0.0736	32.8 ± 5.0	31.9 ± 4.8	0.1743
***Smoking status (%)***						
Never	167 (46.52%)	202 (96.19%)	< 0.0001	73 (51.41%)	96 (94.12%)	Fisher < 0.0001
Ex-smokers	22 (6.13%)	0 (0.00%)		4 (2.82%)	0 (0.00%)	
Current smokers	163 (45.40%)	7 (3.33%)		64 (45.07%)	6 (5.88%)	
Miss	7 (1.95%)	1 (0.48%)		1 (0.70%)	0 (0.00%)	
*Alcohol drinking status (%)*						
Never	66 (18.38%)	162 (77.14%)	*Fisher < 0.0001	26 (18.31%)	74 (72.55%)	Fisher < 0.0001
Ex-drinkers	3 (0.84%)	0 (0.00%)		1 (0.70%)	0 (0.0%)	
Current drinkers	283 (78.83%)	47 (22.38%)		114 (80.28%)	28 (27.45%)	
Miss	7 (1.95%)	1 (0.48%)		1 (0.70%)	0 (0.0%)	
Height (cm)	168.4 ± 6.2	158.0 ± 5.8	< 0.0001	171.3 ± 5.6	160.5 ± 5.6	< 0.0001
Weight (kg)	69.9 ± 10.1	53.7 ± 6.7	< 0.0001	71.7 ± 10.3	53.4 ± 7.0	< 0.0001
BMI (kg/m^2^)	24.6 ± 3.0	21.6 ± 2.6	< 0.0001	24.4 ± 3.2	20.7 ± 2.4	< 0.0001
*Body composition according to BIA*						
BSM (kg)	29.09 ± 3.78	20.23 ± 2.38	< 0.0001	30.22 ± 3.64	20.57 ± 2.58	< 0.0001
BSM/height^2^ (kg/m^2^)	10.24 ± 0.98	8.10 ± 0.72	< 0.0001	10.30 ± 1.01	7.97 ± 0.78	< 0.0001
BFM (kg)	18.26 ± 5.59	16.50 ± 4.50	0.0001	18.17 ± 6.08	15.57 ± 4.13	0.0002
BFM/height^2^ (kg/m^2^)	6.45 ± 1.96	6.64 ± 1.91	0.2607	6.22 ± 2.11	6.06 ± 1.62	0.5226
*Body composition according to CT image*						
SMA (cm^2^)	105.4 ± 16.22	69.78 ± 9.84	< 0.0001	107.9 ± 16.1	71.3 ± 10.5	< 0.0001
SMA/height^2^ (cm^2^/m^2^)	37.11 ± 5.51	27.95 ± 3.77	< 0.0001	36.83 ± 5.54	27.69 ± 3.76	< 0.0001
SMA/weight (cm^2^/kg)	1.51 ± 0.18	1.31 ± 0.17	< 0.0001	1.51 ± 0.17	1.34 ± 0.18	< 0.0001
SMA/BMI (cm^2^/kg/m^2^)	4.29 ± 0.54	3.26 ± 0.50	< 0.0001	4.44 ± 0.53	3.47 ± 0.52	< 0.0001
SMD (HU)^*^	41.46 (6.42)	39.52 (6.97)	< 0.0001	43.66 (5.92)	41.73 (6.33)	< 0.0001
IMAT (cm^2^) ^*^	3.14 (3.52)	2.54 (2.75)	< 0.0001	2.54 (2.78)	1.82 (2.34)	0.002
IMAT/SMA (%) ^*^	3.21 (3.39)	3.47 (4.12)	0.247	2.60 (2.58)	2.47 (2.97)	0.832

Data presented as n (percentage) or mean (standard deviation) if not specified

BMI: body mass index; BFM: body fat mass; BSM: body skeletal muscle; IMAT: intermuscular adipose tissue; SMA: skeletal muscle area; SMD: skeletal muscle radiodensity

^*^ Data presented as median (interquartile range)

### Correlation of body composition indicators measured by CT and BIA

**Fig. 3:**
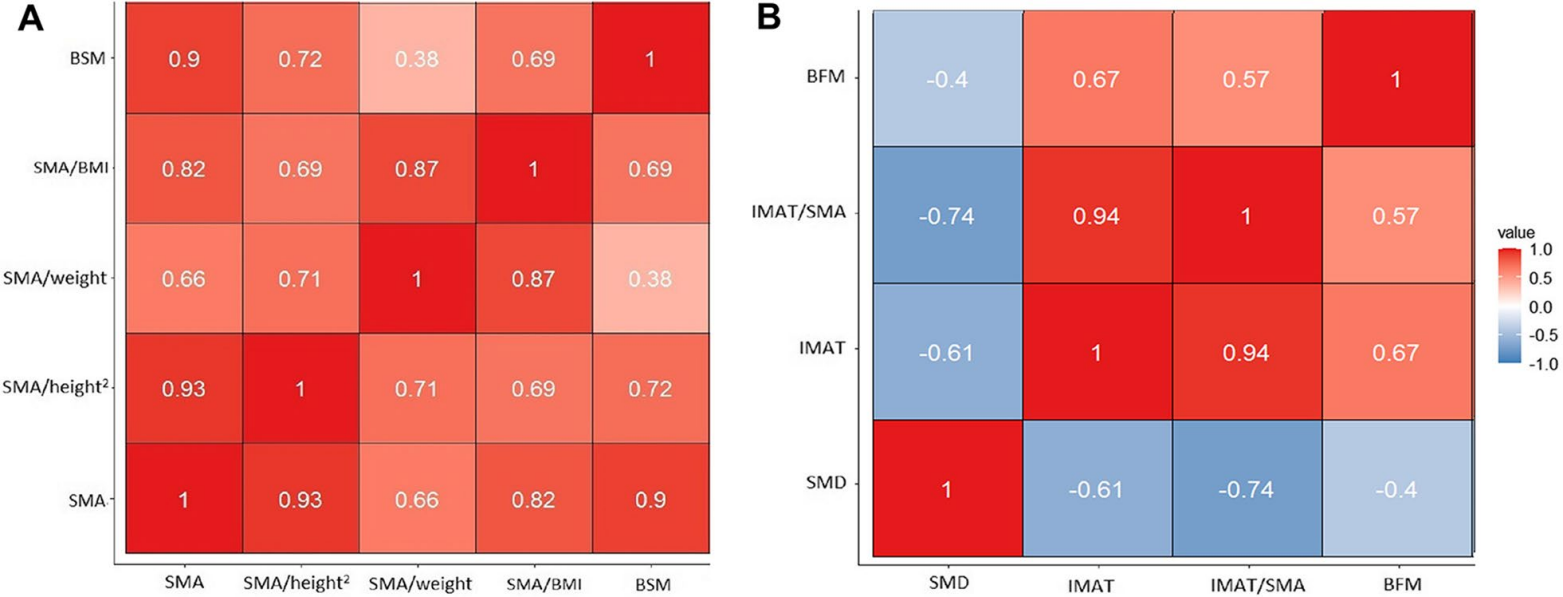
The correlations (Pearson’s r) of SMA and SMIs measured by CT and BSM measured by BIA (**A**) and the correlations (Spearman’s ρ) of SMD, IMAT, and IMAT/SMA measured by CT and BFM measured by BIA (**B**)

Figure 3A shows the correlations (Pearson’s r) of SMA and SMIs measured by CT and BSM measured by BIA; whereas Fig. 3B shows the correlations (Spearman’s ρ) of SMD, IMAT, and IMAT/SMA measured by CT and BFM measured by BIA. As shown in Fig. 3A, SMA, SMA/height^2^, and SMA/BMI were strongly and positively correlated with BSM (*r* = 0.90, 0.72, and 0.69, respectively, all *p* < 0.001); whereas SMA/weight was moderately and positively correlated with BSM (*r* = 0.38, *p* < 0.001). These findings indicated that SMA, SMA/height^2^, and SMA/BMI were the preferred indicators for estimating BSM.

Furthermore, IMAT and IMAT/SMA were strongly and positively correlated with BFM (*r* = 0.67 and 0.58, respectively, both *p* < 0.001), indicating that both IMAT and IMAT/SMA were the preferred indicators for estimating IMAT-defined myosteatosis. Last, SMD was moderately and negatively correlated with BFM (*r* = − 0.40, *p* < 0.001).

**Fig. 4:**
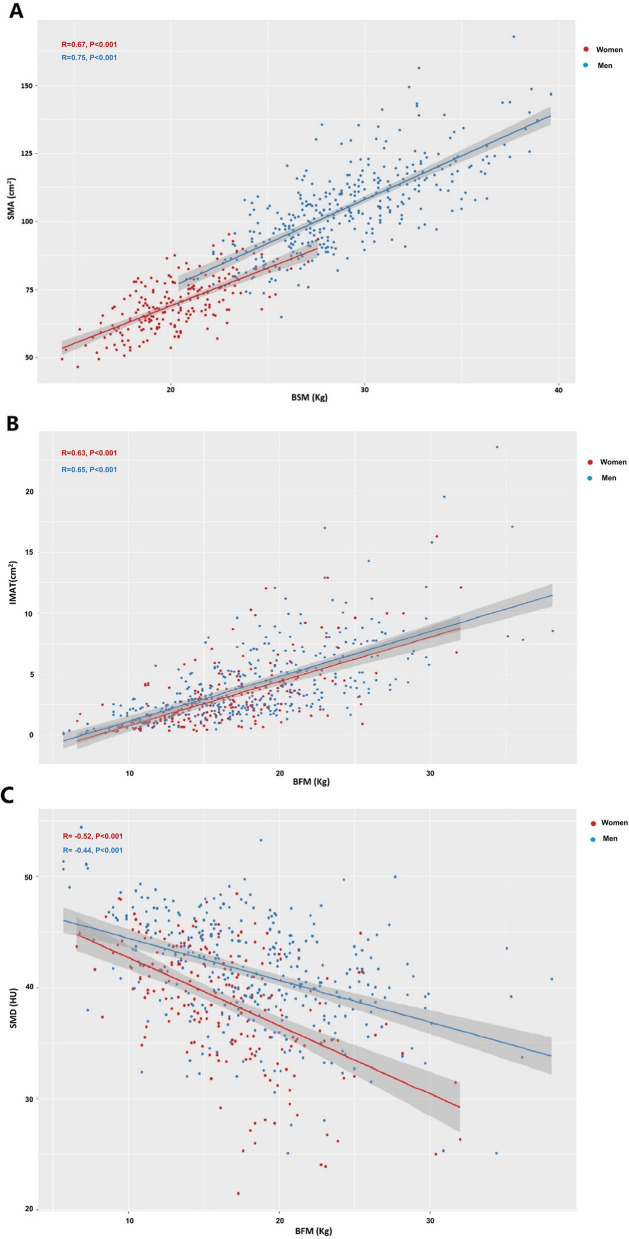
The scatter plots and linear regression lines of SMA with BSM (**A**), IMAT with BFM (**B**), and SMD with BFM (**C**) in men and women

Figure 4 shows the scatter plots and linear regression lines of SMA with BSM (A), IMAT with BFM (B), and SMD with BFM (C) in men and women.

### Reference values for defining sarcopenia and myosteatosis by SMA, SMIs, SMD, IMAT, and IMAT/SMA

Table 2 shows the sex-specific cut-off points of SMA and SMIs equivalent to T-score − 1.0, − 2.0, and − 2.5; SMD equivalent to p1, p5, and p10; and IMAT and IMAT/SMA equivalent to p90, p95, and p99 in the young reference group. When T-score < − 2.0 was used to set the cut-off point for defining sarcopenia, the sex-specific cut-off points of SMA and SMA/height^2^ were < 75.67 cm^2^ and < 25.75 cm^2^/m^2^ in men, and < 50.39 cm^2^ and < 20.16 cm^2^/m^2^ in women, respectively. When p5 was used to set the cut-off point for determining SMD-defined myosteatosis, the cut-off points were < 37.42 HU and < 33.17 HU in men and women, respectively. When p95 was used to set the cut-off point for determining IMAT-defined myosteatosis, the sex-specific cut-off points of IMAT and IMAT/SMA were > 8.72 cm^2^ and > 7.51% in men, respectively, and > 4.58 cm^2^ and > 6.83% in women, respectively.

**Table 2:** Reference data from the young reference group (20–40 years) regarding SMA, SMD, IMAT, and their indices

	Men (n = 142)			Women (n = 102)		
	T score = − 1.0	T score = − 2.0	T score = − 2.5	T score = − 1.0	T score = − 2.0	T score = − 2.5
SMA (cm^2^)	91.76	75.67	67.62	60.85	50.39	45.16
SMA/height^2^ (cm^2^/m^2^)	31.29	25.75	22.98	23.92	20.16	18.28
SMA/weight (cm^2^/kg)	1.34	1.16	1.08	1.17	0.99	0.9
SMA/BMI (cm^2^/kg/m^2^)	3.91	3.37	3.11	2.95	2.43	2.17
	p1	p5	p10	p1	p5	p10
SMD (HU)	33.76	37.42	39.21	29.25	33.17	34.46
	p99	p95	p90	p99	p95	p90
IMAT (cm^2^)	14.35	8.72	7.17	5.28	4.58	4.01
IMAT/SMA (%)	11.19	7.51	6.27	8.99	6.83	6.19

BMI: body mass index; IMAT: intermuscular adipose tissue area; P1: the 1^st^ percentile; P5: the 5^th^ percentile; P10, the 10^th^ percentile; P90: the 90^th^ percentile; P95: the 95^th^ percentile; P99: the 99^th^ percentile; SMA: skeletal muscle area; SMD: skeletal muscle radiodensity

## Discussion

This study demonstrated that T12 SMA and T12 SMA/height^2^ were strongly correlated with BSM; T12 IMAT was strongly correlated with BFM; whereas T12 SMD was moderately correlated with BFM. These findings imply that T12 SMA and T12 SMA/height^2^ may serve as surrogates for diagnosing sarcopenia while T12 SMD and T12 IMAT may serve as surrogates for determining myosteatosis. To the best of our knowledge, this is the first study to report the cut-off points of T12 SMA (and SMIs) for diagnosing sarcopenia, and the cut-off points of T12 SMD and T12 IMAT for diagnosing myosteatosis in Chinese people.

Only two previous studies reported the cut-off points of T12 SMA (and SMIs) measured by chest CT for diagnosing sarcopenia. Both were conducted in Caucasians. Based on a healthy US population, Derstine et al. [20] reported the cut-off points of T12 SMA and SMA/height^2^ for defining sarcopenia were 92.3 cm^2^ and 28.8 cm^2^/m^2^ in men, and 56.1 cm^2^ and 20.8 cm^2^/m^2^ in women, respectively. Based on the Michigan Kidney Donor Candidate Population, the same team reported the cut-off points of T12SMA and SMA/height^2^ for defining sarcopenia were 91.5 cm^2^ and 28.7 cm^2^/m^2^ in men, and 55.9 cm^2^ and 20.6 cm^2^/m^2^ in women, respectively [21]. As expected, the T12 SMA in the US population was higher than that in our study population in both men and women. However, after adjusting for body size, the T12 SMA/height^2^ was very similar across the three study populations. This finding supports the utility of T12 SMA/height^2^ (rather than SMA) to define sarcopenia to facilitate the comparability between studies.

Notably, there remains an unsolved controversy regarding the definition of sarcopenia [11]. In brief, most researchers in the field of oncology and surgery define sarcopenia as low muscle mass per se [22]. For example, the North American Expert Opinion Statement on Sarcopenia in Liver Transplantation recommends defining sarcopenia “using only muscle mass” [23]. In contrast, in the field of geriatrics and gerontology, a consensus has been achieved that the definition of sarcopenia should include not only low muscle mass but also low muscle strength and/or low physical performance [2, 24]. Traditionally, CT-defined sarcopenia was widely studied in oncology and surgery because opportunistic CT images could easily be obtained from patients with cancer or having surgery [11]. However, studies regarding CT-defined sarcopenia in older adults are emerging [25, 26].

Myosteatosis, as an important indicator of muscle quality, has increasingly been attracting researchers’ attention in the field of skeletal muscle aging [5]. The diagnosis methods of myosteatosis have not been established yet; however, IMAT and SMD were the two most frequently used indicators to define myosteatosis in the literature [5]. According to a recent systematic review, among the 125 included studies, 82 (65.6%) and 27 (21.6%) studies used IMAT and SMD to define myosteatosis, respectively [11]. Besides, the abdomen and thigh were the most frequently scanned body positions in the literature. In this same systematic review, 61 (48.8%) and 48 (38.4%) studies used abdominal CT images and thigh CT images, respectively. However, no study defined myosteatosis based on chest CT [11]. Our study provided the cut-off points of T12 SMD (men: < 37.42 HU; women: < 33.17 HU) and T12 IMAT (men: > 8.72 cm^2^; women: > 4.58 cm^2^) for defining myosteatosis base on chest CT. This method can be used to define myosteatosis in Asian populations. However, these cut-off points need to be further validated in different populations.

Which one is better for defining myosteatosis, SMD or IMAT? There is currently no answer to this question. However, a recent study indicated that SMD (but not IMAT) was independently associated with peak knee extension torques and rates of torque development in older adults [27]. In a retrospective study conducted in ICU patients, higher SMD was significantly associated with lower 6-month mortality; whereas higher IMAT was not significantly associated with higher 6-moth mortality after adjustment for confounders [28]. Furthermore, it has been reported that both SMD and IMAT were associated with mortality in men; but only SMD (not IMAT) was associated with mortality in women [5]. More prospective studies are needed to answer this question.

MRI is supposed to be the gold standard to analyze body composition, especially myosteatosis. MRI is a more sensitive tool for detecting muscle fat diffusion and visualizing anatomical structures than CT [29, 30]. Additionally, MRI can analyze the composition and detailed structure of individual muscles, assisting in the differentiation of edema, fatty infiltration, and fibrosis [29, 30]. Furthermore, magnetic resonance spectroscope (MRS) can precisely differentiate IMCL, IntraMAT, and IMAT [31]. Both MRI and MRS do not expose patients to radiation. However, MRI and MRS are very expensive and not readily accessible, which limits their use in clinical research and practice as tools to identify sarcopenia and myosteatosis [31].

### Clinical implications

Compared with BIA and dual-energy x-ray absorptiometry (DXA), CT is limited because it exposes the participants to radiation. Therefore, muscle mass and muscle quality measurements are commonly analyzed based on clinically acquired CT scans for the management of other diseases [11]. In clinical practice, chest CT scans are far more frequently used than abdominal CT and thigh CT scans. In China, chest CT scans have been widely used in routine health examinations for screening lung cancer and other diseases in middle-aged and older adults. Therefore, the opportunistic utility of chest CT images would provide a greater opportunity to identify other diseases, such as chronic obstructive pulmonary disease [32] and osteoporosis [33]. Our study implies that the opportunistic utility of chest CT images is also useful for identifying sarcopenia and myosteatosis.

Notably, our study segmented SMA and IMAT in the CT images based on the most widely accepted HU thresholds (SMA: − 29 to 150 HU; IMAT: − 30 to − 190 HU) according to the recent systematic reviews [11, 34]. Therefore, the cut-off points for the muscle mass and muscle quality indicators provided in our study may not be suitable for the studies that apply other HU thresholds to define skeletal muscle mass or IMAT (e.g., 0 to 100 HU to define muscle mass as previously reported).

### Limitations

First, the participants of this study were participants in routine health examinations at the health management center of a tertiary hospital, which is the largest hospital in western China that provides comprehensive health examinations for individuals from western China. Because the participants were voluntarily joined in this study during routine health examinations, the representation of our study population may be limited. Therefore, the generalization of our results to other populations should be cautious. Second, we estimated the BSM and BFM with BIA instead of DXA. However, a recent study found that DXA and segmental multi-frequency BIA (InBody 770, the exact device type that we used in this study) were comparable for estimating BSM and BFM [35]. Third, it remains unclear whether the cut-off points of muscle mass and muscle quality indicators that we proposed in this study can predict outcomes or not. We have conducted a prospective cohort study in patients with advanced non-small-cell lung cancer to validate our results.

## Conclusion

Muscle mass indicators (SMA and SMIs) and muscle quality indicators (SMD, IMAT, and IMAT/SMA) measured by chest CT images at the T12 level could be used for diagnosing sarcopenia and myosteatosis, respectively. We suggest SMA/height^2^ (< 25.75 cm^2^/m^2^ in men and < 20.16 cm^2^/m^2^ in women) for diagnosing sarcopenia and SMD (< 37.42 HU in men and < 33.17 HU in women) or IMAT (> 8.72 cm^2^ in men and > 4.58 cm^2^ in women) for diagnosing myosteatosis in Chinese people. However, the cut-off points of these indicators need to be further validated in different populations.

## Data Availability

The datasets used and/or analyzed during the current study are available from the corresponding author on reasonable request.

## Abbreviations

BFM: Whole-body fat mass
BIA: Bioelectrical impedance analysis
BMI: Body mass index
BSM: Whole-body skeletal muscle mass
CT: Computed tomography
EWGSOP2: The updated version of the European Working Group on Sarcopenia in Older People
HDL-C: High-density lipoprotein-cholesterol
HU: Hounsfield Unit
IMAT: Intermuscular adiposity tissue
IMCL: Intramyocellular lipids
IntraMAT: Intramuscular adipose tissue
L3: The 3^rd^ lumbar vertebra
LDL-C: Low-density lipoprotein-cholesterol
SMA: Skeletal muscle area
SMD: Skeletal muscle radiodensity
SMI: Skeletal muscle index
T12: The 12^th^ thoracic vertebra
WC: Waist circumference
